# Decoding iron deficiency in cancer: mechanisms, immune modulation, and therapeutic potential

**DOI:** 10.3389/fnut.2025.1650929

**Published:** 2025-10-20

**Authors:** Lihao Lin, Xunzhe Yin, Yi Guan

**Affiliations:** ^1^Department of Neurosurgery, First Hospital of Jilin University, Changchun, China; ^2^Center for Theoretical Interdisciplinary Sciences, Wenzhou Institute, University of Chinese Academy of Sciences, Wenzhou, China

**Keywords:** iron deficiency, tumor progression, immune response, tumor metabolism, therapeutic targets

## Abstract

Iron is a vital micronutrient in many biological functions, including DNA metabolism, oxygen transport, and cellular energy generation. In this context, it is intimately linked to cancer biology. However, although many studies have comprehensively investigated and reviewed the effects of excess iron on tumor initiation and progression, the potential interrelations of iron deficiency with tumors have been largely neglected and need to be better defined. Recent studies have highlighted the complex relationship between iron deficiency and tumor biology. Iron deficiency in specific tumor types can promote tumor progression through activation of hypoxic responses, metabolic reprogramming, and suppression of the immune response, as well as inhibit tumor growth by limiting tumor cell proliferation, among other mechanisms. This review aims to systematically explore the dual mechanisms of iron deficiency in tumors, its specific effects in different tumor types, its impact on tumor metabolism, immune responses, and therapy, and its prospects as a potential therapeutic target. Furthermore, the potential of iron metabolism markers in tumor diagnosis and prognosis is discussed. By synthesizing existing evidence, this paper comprehensively explains how iron deficiency affects tumorigenesis and identifies future research and clinical practice directions.

## 1 Introduction

Iron is an essential trace element vital for maintaining human health (1, 2). It is critical in oxygen transport, DNA synthesis, energy metabolism, and immune regulation (1, 3). Primarily, iron serves as a core component of hemoglobin, supporting oxygen delivery to tissues, and is also involved in myoglobin, cytochromes, and various enzymes (2, 4). The precise regulation of iron metabolism is essential for maintaining iron homeostasis. Iron deficiency (ID), a widespread nutritional deficiency, is especially prevalent among high-risk groups, including pregnant women, young children, and the elderly. These populations are particularly vulnerable to ID, which can have significant health consequences (5). According to the 2018 ESMO guidelines, ID is defined by ferritin (FTN) levels below 100 μg/L (absolute iron deficiency, AID) or FTN levels ≥100 μg/L with a transferrin saturation (TSAT) under 20% (functional iron deficiency, FID) (6, 7). Typical symptoms of ID include pallor, cold skin, fatigue, restless leg syndrome, reduced physical performance, brittle nails, dry lips, cognitive impairment, headaches, insomnia, depression, decreased libido, shortness of breath, tachycardia, thrombocytosis, increased risk of thromboembolic complications, and hair loss (8).

While numerous studies have extensively examined the effects of iron overload on cancer initiation and progression, the potential relationship between ID and cancer has been largely overlooked and remains poorly defined. However, growing interest has been in exploring the potential link between ID and tumor progression. ID not only impacts the overall health of cancer patients but may also directly influence cancer development and progression (1). Studies indicate that the prevalence of ID among cancer patients is 57.9%, although the exact proportions of FID and AID have not been entirely determined (9, 10). ID prevalence is notably higher in women than in men, with significantly elevated rates in premenopausal women (76.0%) compared to postmenopausal women (61.9%). This disparity is primarily due to physiological blood loss—particularly from menstruation—as well as increased iron demands during pregnancy and lactation. Moreover, women often have a relatively lower dietary intake of bioavailable heme iron, all of which contribute to reduced iron stores and a higher risk of ID. Additionally, 36% of cancer patients with ID present with anemia, with approximately half of these anemic patients having hemoglobin levels between 10 and 11 g/dl (9). Anemia, defined as hemoglobin (Hb) < 12 g/dl, was observed in 67% of cancer patients undergoing chemotherapy during a 6-month surveillance period. Radiotherapy patients are also at risk: the prevalence of anemia increased from 34.8% at baseline to 38.9% by the end of radiotherapy, with 17.5% of patients developing new-onset anemia during treatment (8, 11). In esophageal squamous cell carcinoma patients, approximately 29.1% were anemic during primary radiotherapy, and anemia was associated with significantly poorer overall and disease-free survival outcomes (12). Among anemic patients, the prevalence of ID was 64.8%, while 54.1% of non-anemic patients also showed evidence of ID (9). In summary, the prevalence of ID in cancer patients is high, regardless of the presence of anemia.

Iron's dual role in cancer development has become a topic of considerable interest in recent research. On one hand, ID may inhibit tumor cell growth, as iron is essential for cellular proliferation and metabolism. On the other hand, low iron levels may lead to compensatory adaptations in the tumor microenvironment (TME), such as increased hypoxia and inflammation, which can drive tumor cells to become more aggressive (13). Furthermore, systemic anemia, immunosuppression, and alterations in oxidative stress induced by ID may also have complex effects on tumor progression (14). Current research has uncovered mechanisms suggesting that disruptions in iron metabolism are closely linked to the progression of various cancers. Studies have identified a U-shaped relationship between iron intake and cancer risk, indicating that both high and low iron intake levels are associated with increased cancer risk (15). For instance, tumor cells, which have a higher iron demand than normal cells, often increase iron uptake by upregulating iron transporter proteins or downregulating iron storage proteins. Furthermore, the accumulation of excess iron can contribute to tumorigenesis by fostering the production of reactive oxygen species (ROS), which in turn can cause DNA damage. However, the specific mechanisms by which ID influences cellular processes in tumor biology remain poorly understood, representing a crucial gap in current research. This review aims to systematically summarize the mechanism of ID in tumor progression and explore its biological effects in tumorigenesis, proliferation, metastasis, and regulation of the tumor microenvironment. In addition, this review will discuss the relationship between ID and prognosis, treatment response, and immune regulation in cancer patients. By summarizing the existing literature, this review explores the promise of iron metabolism as a potential target for cancer therapy and hopefully provides directions for future cancer therapy research.

We conducted a comprehensive literature search in PubMed to identify relevant studies on ID and cancer. The search used the keywords “iron deficiency” AND (“cancer” OR “tumor”) and covered publications from 1990 to 2025. Inclusion criteria were: (1) original research articles, reviews, or meta-analyses published; (2) studies directly addressing the relationship between ID and tumor biology, prognosis, or treatment; (3) availability of full-text. Exclusion criteria were: (1) case reports, conference abstracts, or editorials; (2) studies unrelated to ID in cancer; (3) articles lacking sufficient methodological details. Following initial screening of titles and abstracts, full texts of potentially eligible studies were reviewed to confirm inclusion. Additionally, during manuscript preparation, targeted searches were performed to retrieve articles providing mechanistic explanations or supplementary information relevant to specific sections of the review.

## 2 Biological functions and metabolic mechanisms of iron

### 2.1 Physiological functions and metabolic regulation of iron

Iron serves numerous essential functions in normal cells, supporting energy production, oxygen metabolism, DNA synthesis, and molecular synthesis. It is a required element for various enzymes (Table 1). Most iron exists within heme proteins, such as hemoglobin and myoglobin, forming the central atom of heme—a porphyrin structure partly bound to proteins. Among these proteins, iron is not only responsible for oxygen transport and storage but also promotes oxygen metabolism through oxidases, peroxidases, and catalase, among others. Additionally, cytochromes within heme proteins are vital to the mitochondrial electron transport chain, ensuring efficient energy production.

**Table 1:** Impact of ID on key iron-dependent enzymes.

**Categorization**	**Designation**	**Main functions**	**Effects of ID**	**Biological consequences**	**Role in the TME**
Oxidoreductase	Cytochrome oxidase (complex IV)	Key enzyme in the mitochondrial electron transport chain that generates ATP	Reduced electron transfer efficiency	Decreased ATP production affects the function of high-energy demand cells	Inhibits tumor cell energy production and may affect tumor proliferation
	Catalase	Breakdown of hydrogen peroxide to prevent oxidative stress	Hydrogen peroxide accumulation	Increased oxidative stress leading to DNA damage and inflammatory responses	Increased oxidative stress in the TME may promote inflammation and tumor progression
Fe-sulfur cluster enzyme	Ribonucleotide reductase	Reduction of ribonucleotides to deoxyribonucleotides for DNA synthesis	Decreased DNA precursor production	DNA synthesis is inhibited, affecting cell proliferation and repair	Limiting the proliferation of tumor cells and T cells and weakening the anti-tumor immune response
	NADH dehydrogenase	Electrons are transferred in the electron transport chain, driving the oxidative phosphorylation process	Electron transport blockage	Decreased ATP production, affecting energy production	Inhibits tumor cell metabolism and limits tumor growth
Porphyrin and heme synthases	ALA synthase	Heme synthesis rate-limiting enzyme that produces hemoglobin and cytochromes	Reduced activity, inhibition of heme synthesis	Reduced hemoglobin, limited oxygen transport, impaired mitochondrial function	Impacts oxygen supply and mitochondrial metabolism in the TME and may promote hypoxia
	Heme oxygenase	Breaks down hemoglobin, releasing iron and carbon monoxide	Impaired heme degradation, affecting iron cycling and antioxidant functions	Affects iron metabolism homeostasis and may trigger cellular hypoxia	Increases tumor hypoxia and facilitates tumor adaptation to unfavorable environments
Lipoxygenase and lipid metabolizing enzymes	Lipoxygenase	Catalyzes the oxidation of arachidonic acid to generate lipid signaling molecules	Decreased lipid peroxidation	Inhibition of lipid peroxidation affects cell membrane stability and ferroptosis processes	Inhibition of the ferroptosis pathway may promote tumor cell survival
	Cyclooxygenase	Involved in prostaglandin synthesis, regulation of inflammation and blood flow	Decreased prostaglandin production	Suppresses inflammatory responses and may affect immune regulation	Reduced immune response and weakened immune clearance
Antioxidant enzyme	SOD	Reduces oxidative stress by converting superoxide to hydrogen peroxide	Superoxide accumulation	Increased oxidative stress, damage to cell membranes and DNA	Enhanced oxidative stress facilitates tumor cell adaptation and survival
	GPX4	Decompose lipid peroxides to prevent membrane damage	Indirectly limited, increased lipid peroxidation	Increased oxidative stress and cell membrane damage	Impacts antioxidant defenses of tumor cells and promotes cell survival
DNA repair and synthesis enzymes	DNA polymerase	Involved in DNA replication and repair	Limited activity and decreased DNA repair efficiency	DNA damage accumulates and affects genome stability	Increases tumor mutation rate and promotes tumor progression
	Topoisomerase	Unwinding the DNA superhelix to facilitate replication and transcription	Structural damage and reduced catalytic efficiency	Reduced DNA stability, prone to mutations	Promoting genetic variation in tumors and increasing tumor diversity
Complement-related enzyme	Complement C3 convertase	Labeling and removal of pathogens and damaged cells in the complement system	Weakened complement function	Reduced pathogen clearance efficiency and weakened innate immune function	Attenuating immune surveillance

Iron is essential for several critical metabolic and synthetic processes in non-heme proteins. For example, iron-sulfur clusters and mitochondrial acetylases are vital players in the electron transport chain and energy metabolism, supporting cellular metabolic activity. Iron is also a core component of ribonucleotide reductase, an enzyme necessary for DNA precursor synthesis; thus, ID inhibits DNA synthesis, limiting cell proliferation and repair (4). Beyond these roles, iron in non-heme proteins contributes to synthesizing biomolecules like collagen, tyrosine, and catecholamines, which are vital for maintaining cellular structure and signal transduction (8, 16).

Iron acts as a cofactor for cytochrome complexes in the cellular respiratory chain in normal cells, facilitating oxygen reduction and energy production. Cytochrome c oxidase, in particular, plays a crucial role in mitochondrial ATP synthesis. ID can impair mitochondrial function, reducing energy production and compromising cell function (17). Additionally, in the context of oxidative stress, iron participates in the Fenton reaction, where excess free iron can induce oxidative stress and DNA damage (18, 19). Under normal physiological conditions, iron toxicity is controlled by its binding to proteins, which prevents the harmful effects associated with free iron.

**Figure 1:**
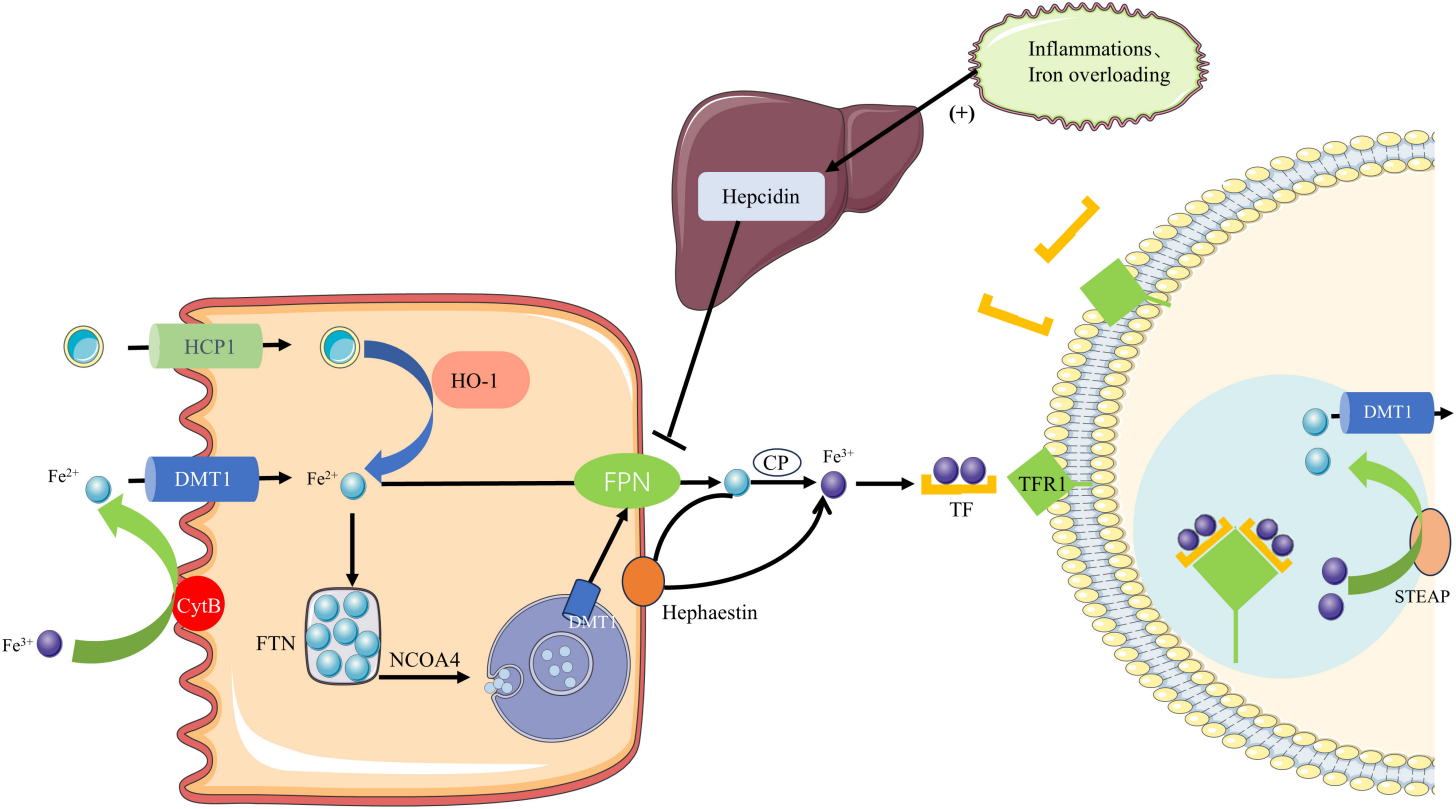
Iron homeostasis regulation. Iron is absorbed in the intestine as heme or non-heme iron. Heme iron enters enterocytes via HCP1 and is degraded by HO-1 to release Fe^2+^. Non-heme iron is reduced to Fe^2+^ by DCYTB and imported via DMT1. Fe^2+^ can be stored in FTN or exported through FPN1 after oxidation by hephaestin or ceruloplasmin. Under iron deficiency, ferritin is degraded via NCOA4-mediated autophagy to release Fe^2+^, which is transported out via lysosomal DMT1 and FPN1. In plasma, TF binds Fe^3+^ and delivers it to tissues through TFR1–mediated endocytosis. Fe^3+^ is reduced by STEAP in endosomes, and Fe^2+^ is released into the cytoplasm via DMT1, while TF-TFR1 is recycled to the membrane.

Iron is absorbed in the intestine in two forms: heme and non-heme iron. Heme iron is taken up by heme carrier protein 1 (HCP-1) on the apical surface of enterocytes. Once inside the enterocytes, heme oxygenase-1 (HO-1) extracts ferrous iron from the heme, releasing it into the cytoplasm (Figure 1). In contrast, non-heme iron must first be reduced to Fe^2+^ by the iron reductase cytochrome B on the apical membrane before being absorbed into enterocytes via the divalent metal transporter 1 (DMT1). Fe^2+^ is stored in enterocytes as part of FTN when body iron levels are adequate or high. In conditions of low body iron, Fe^2+^ is mobilized into circulation through two pathways: either directly exported through ferroportin-1 (FPN1) on the basal membrane, where it is oxidized to Fe^3+^ by copper-containing iron oxidases, such as ceruloplasmin and hephaestin, or by FTN degradation via autophagy mediated by nuclear receptor coactivator 4 (NCOA4). In the latter pathway, NCOA4 interacts with FTN and transports it to autophagosomes, which subsequently fuse with lysosomes. Within the lysosomes, FTN undergoes degradation, releasing iron into the cytoplasm through lysosomal DMT1. From there, the iron can be exported into circulation via FPN1 (20, 21).

Transferrin (TF), the primary iron transport protein in plasma, transports iron to tissues where it binds to transferrin receptors (TFR1) on the cell surface. Each transferrin molecule can bind two Fe^3+^ ions, forming TF-(Fe^3+^)_2_ complexes that bind to TFR1 and are internalized into endosomes. In the endosome, Fe^3+^ is reduced to Fe^2+^ by STEAP, after which Fe^2+^ is transported to the cytoplasm through DMT1, while the TF-TFR1 complex recycles back to the cell membrane for subsequent iron transport cycles. Iron homeostasis is maintained by the coordinated regulation of these critical proteins (21).

### 2.2 Abnormal iron regulation in tumors

Although there is considerable variation between cancer types, the expression of proteins involved in iron uptake, storage, and export is consistently altered in tumors at the cellular level (1). The regulation of iron homeostasis plays a critical role in tumor progression and involves complex interactions of multiple signaling molecules and proteins. Under normal physiological conditions, the body maintains iron homeostasis by regulating core molecules such as hepcidin, FPN, and TFR. However, in the TME, these regulatory mechanisms are often perturbed, leading to abnormalities in iron metabolism that support tumor cell growth and proliferation.

Iron metabolism varies significantly between normal, peripheral, and tumor tissues, reflecting the distinct regulatory mechanisms in cancer cells. In normal and peripheral tissues, iron levels are primarily determined by systemic iron homeostasis. However, tumor tissues often exhibit an altered iron metabolism characterized by increased iron uptake and retention, a phenomenon known as “iron addiction.” Many malignancies, such as breast cancer, prostate cancer, and hepatocellular carcinoma, achieve this by upregulating TFR1 to enhance iron uptake while downregulating FPN to reduce iron export, ensuring a continuous supply of iron to support rapid tumor proliferation and metabolic reprogramming (1, 13).

Interestingly, the relationship between systemic ID and tumor iron levels is not uniform across all cancers. While some tumors maintain iron homeostasis through enhanced iron sequestration, others, such as colorectal cancer, pancreatic cancer, and certain brain tumors, may experience local ID due to poor vascularization, hypoxia, and ischemia. These microenvironmental constraints can limit iron availability, activating adaptive pathways such as HIF-1α, which promotes metabolic adaptation and tumor invasiveness (3, 22). This heterogeneity underscores the complexity of iron regulation in cancer and highlights the need for precision in iron-targeted therapeutic strategies.

Due to their rapid growth and proliferation, tumor cells have an increased demand for iron, disrupting iron metabolism. Iron uptake in tumor cells primarily relies on TFR1 and DMT1 (1). TFR1 is a vital protein mediating iron uptake, which introduces iron into cells by binding to transferrin carrying Fe^3+^. Many tumor types, such as breast, lung, and liver cancers, are usually accompanied by high expression of TFR1, and this up-regulation of expression is closely associated with tumor aggressiveness and poor prognosis (23–27). High expression of TFR1 enables tumor cells to acquire iron more efficiently in a low-iron environment to meet the metabolic demands required for their rapid proliferation. In addition, it has been found that some novel drugs targeting TFR1 can inhibit tumor growth by blocking iron uptake by tumor cells, which opens up new possibilities for cancer treatment strategies. DMT1, which plays a key role in both intestinal iron absorption and the transport of iron from endosomes to the cytoplasm, is significantly upregulated in colorectal cancer (1, 28).

Recent evidence indicates that, beyond TFR1 and DMT1, lipocalin-2 (LCN2, also called neutrophil gelatinase-associated lipocalin or NGAL) provides an alternative iron-delivery pathway that is exploited by multiple tumor types. LCN2 is a secreted siderophore-binding protein that can capture iron-containing complexes in iron-poor niches and deliver iron to cancer cells via specific receptors (e.g., SLC22A17/24p3R), thereby supporting tumor cell survival and proliferation under limiting-iron conditions (29). This mechanism has been directly demonstrated in leptomeningeal metastasis models and is supported by clinical specimens showing tumor-associated LCN2 expression (29). Moreover, LCN2 is frequently upregulated in diverse cancers and has been linked to EMT, invasion, angiogenesis, and modulation of the tumor microenvironment, including interactions with myeloid cells and neutrophils (30, 31). Its net effect integrates both iron delivery and pleiotropic signaling functions that promote malignancy. Taken together, these data argue that LCN2 represents an important, tumor-exploited iron trafficking axis that complements canonical TFR1/DMT1 routes and should be considered when assessing tumor iron biology and designing iron-targeted interventions.

Hepcidin, an essential hormone regulating systemic iron homeostasis, is a hepatic peptide widely regarded as a primary iron regulator (32). By binding to FPN, Hepcidin inhibits iron release from intestinal epithelial cells and macrophages, thereby controlling iron absorption and distribution within the body (33). Serum hepcidin levels are elevated in many cancers, including hepatocellular carcinoma, prostate, breast, multiple myeloma, ovarian cancers, non-Hodgkin lymphoma, and gastrointestinal cancers (34–40). Moreover, certain cancer cells, including those in breast, colorectal, and prostate tumors, secrete elevated levels of hepcidin compared to their non-malignant counterparts (40–43).

Pro-inflammatory cytokines—such as IL-6, IL-1, TNF-α, and IFN-γ–can further upregulate hepcidin, disrupting the FPN/Hepcidin pathway and inhibiting iron export, thus affecting iron levels in the body. This dysregulation reduces serum iron and increases FTN stores, leading to a state of FID commonly observed in cancer patients and constituting a significant mechanism of cancer-related ID (13). The prevalence of FID increases with cancer stage and correlates with poorer treatment outcomes in cancer patients. Additionally, the HFE-TFR2 complex forms a multiprotein structure that activates SMAD1/5/8 via HJV/BMP phosphorylation, further promoting Hepcidin synthesis (44).

FPN is the only known cellular iron efflux protein, and its expression level plays a crucial role in regulating intracellular iron levels in tumor cells, which were found to be widely down-regulated in various tumors (33). Levels of FPN-1 are significantly reduced in breast, prostate, and liver cancer cells compared with normal cells (41, 42, 45). In breast cancer, lower FPN-1 expression is associated with decreased metastasis-free and disease-specific survival, serving as a predictor of patient outcomes (40, 41). Hypoxia in the tumor microenvironment further regulates iron metabolism. Hypoxia also influences iron regulation by inactivating cytoplasmic prolyl hydroxylase domain (PHD) proteins, for which iron acts as a necessary cofactor. Under hypoxic conditions, PHD enzymes are inactivated, stabilizing HIF-1 and HIF-2, which then translocate to the nucleus. Nuclear translocation of HIF-2α leads to the activation of target genes, including the iron uptake genes TFR1 and DMT1 in enterocytes (21). This coordinated mechanism results in increased systemic iron uptake and enhanced erythropoiesis.

Genetic alterations in JHDMs [Jumonji-C (JmjC) domain-containing histone demethylases] have been identified in various human cancers. JHDMs influence gene expression by demethylating lysine residues on histone tails, a primary site for post-translational modifications (22). JHDMs are iron-dependent enzymes that require iron as a cofactor; thus, ID may inhibit JHDM activity and potentially promote tumorigenesis (22, 46).

ID may also affect miRNA biogenesis and expression through multiple mechanisms, altering the miRNA-mediated gene regulatory network. First, ID is thought to disrupt heme biosynthesis and degradation, impacting cellular iron homeostasis. Additionally, ID may lead to tissue hypoxia and elevated ROS levels, further interfering with miRNA production and regulatory mechanisms. These changes in miRNA expression can have widespread effects on gene regulatory networks, subsequently influencing cellular functions and metabolic pathways (47).

Additionally, the iron storage mechanisms in tumor cells are often dysregulated. Certain tumors maintain high free iron levels by downregulating FTN expression, which supports their growth (48). Oxidative stress driven by iron dependency is also essential in promoting tumor progression, as excess iron promotes DNA damage, cancer cell proliferation, metastasis, and chemotherapy resistance through increased ROS production (23, 49).

## 3 Effect of ID on tumor cell proliferation

Iron is vital for the rapid growth of tumor cells due to its key roles in DNA synthesis, metabolic processes, and energy production. In ID conditions, tumor cell proliferation is generally expected to be inhibited. This is because iron-dependent enzymes are critical for DNA synthesis, and ID reduces the activity of these enzymes, thereby hindering cell division and growth. Additionally, ID may inhibit protein synthesis by downregulating the mTOR signaling pathway, further limiting tumor cell proliferation (50).

**Figure 2:**
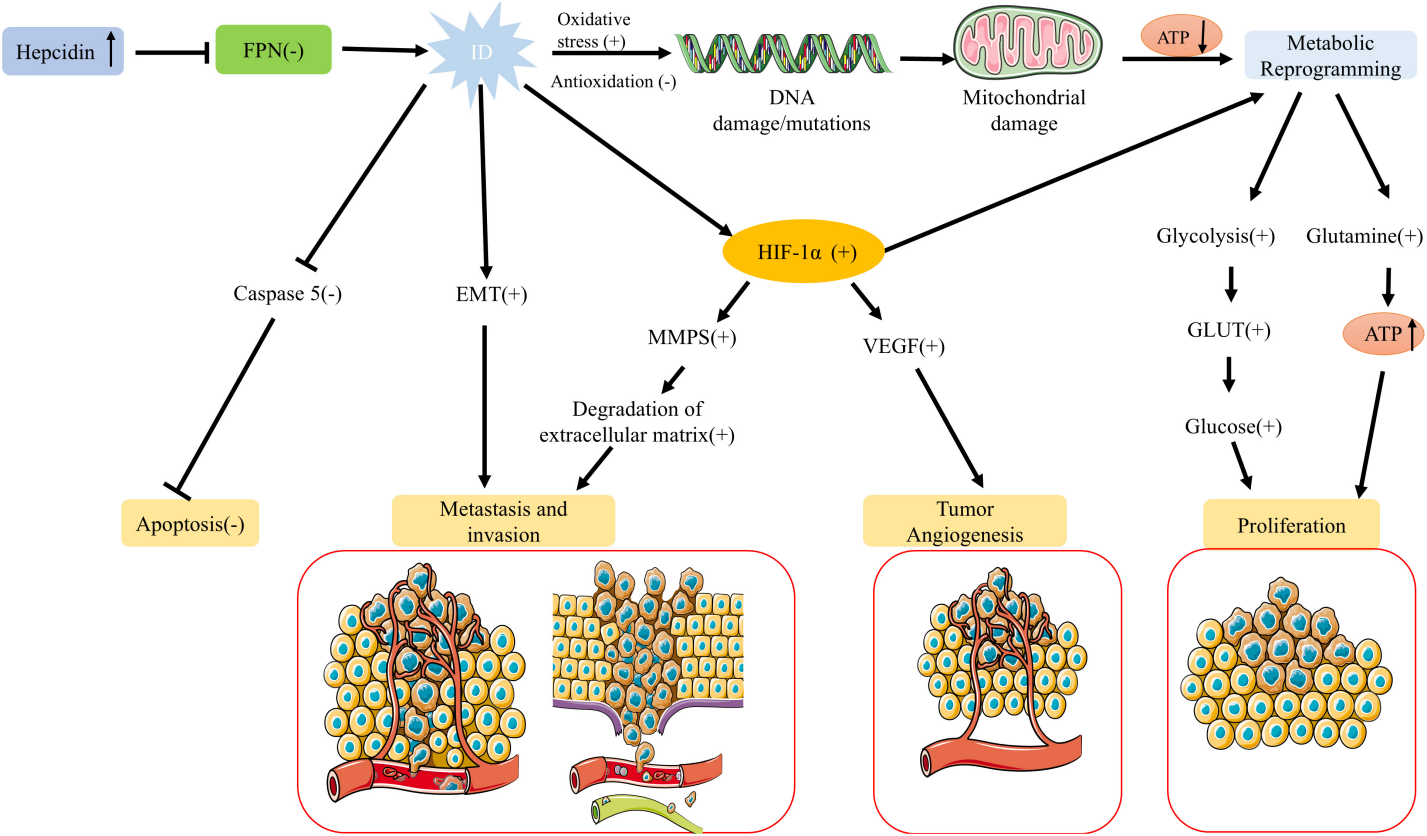
Regulation of tumor growth under ID. While ID generally suppresses tumor cell proliferation, it can also promote tumor survival and progression under specific conditions. ID stabilizes HIF-1α, enhancing glycolysis, inducing VEGF-mediated angiogenesis, and facilitating adaptation to hypoxic, low-iron environments. HIF-1α activation also upregulates MMPs, promoting extracellular matrix degradation, migration, and invasion. ID can induce EMT in various cancers, increasing metastatic potential, and may enhance anti-apoptotic capacity by downregulating caspase 5 expression. Together, these adaptations support tumor survival, invasion, and metastasis in low-iron conditions.

However, recent studies indicate that ID does not permanently inhibit tumor cell proliferation. Under certain conditions, it may instead support tumor cell survival and progression (Figure 2). For example, ID can facilitate tumor cell adaptation to adverse microenvironments by stabilizing HIF-1α. HIF-1α activation not only promotes metabolic reprogramming in tumor cells (e.g., enhancing glycolysis) but also induces vascular endothelial growth factor (VEGF) expression, leading to new blood vessel formation that supplies additional oxygen and nutrients to tumor cells (51). This adaptive mechanism enables tumor cells to survive and proliferate in low-iron, hypoxic conditions. Furthermore, HIF-1α activation is closely associated with tumor migration and invasion, as it can upregulate matrix metalloproteinases (MMPs) and other proteins, facilitating extracellular matrix degradation and increasing tumor cells' invasive and metastatic potential (52).

Studies have shown that a low-iron environment can induce epithelial-mesenchymal transition (EMT), enhancing tumor cells' migratory and invasive abilities and increasing their metastatic potential. This phenomenon has been observed in multiple cancer types, including hepatocellular carcinoma and breast cancer (22, 44, 53). Additionally, other studies suggest that ID may enhance the anti-apoptotic capacity of tumor cells. ID downregulates the cysteine protease cathepsin Z gene, associated with cancer and inflammation, and inhibits the expression of caspase 5, a cysteine protease involved in cell apoptosis (22, 54). Thus, while ID typically inhibits tumor cell proliferation, it may also enhance tumor cell survival, invasion, and metastasis by activating adaptive signaling pathways and modifying the tumor microenvironment under specific conditions.

Several *in vitro* studies support that ID can promote aggressive tumor phenotypes. In MDA-MB-231 breast cancer cells, deferoxamine (DFO) increased HIF-1α levels and enhanced migration and invasion in wound-healing and transwell assays (55). In colorectal cancer models, iron chelation induced EMT-like changes and increased invasive behavior (56). In triple-negative breast cancer cells, DFO altered mitochondrial iron distribution, raised ROS and activated NF-κB/TGF-β signaling, leading to greater migration (57). Cancer cells can also upregulate lipocalin-2 and its receptor to scavenge scarce iron, sustaining growth in iron-poor niches (29). These findings provide direct experimental support for the pro-progression effects of ID discussed above.

## 4 Effects of ID on tumor cell metabolism

**Figure 3:**
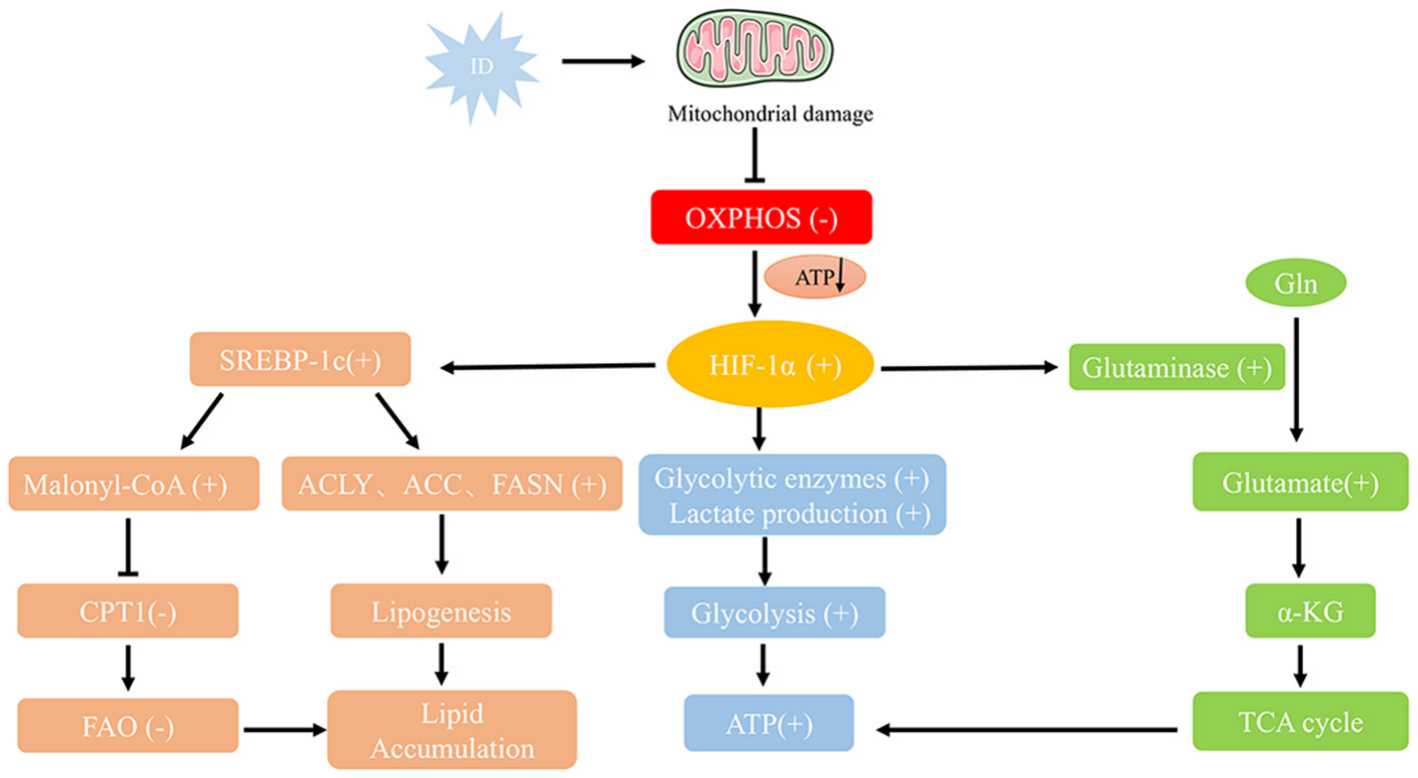
Metabolic reprogramming pathways under ID conditions. ID impairs mitochondrial function by increasing oxidative stress and damaging mitochondrial DNA, reducing oxidative phosphorylation and ATP production. Stabilized HIF-1α shifts metabolism toward glycolysis by upregulating glycolytic enzymes and lactate production, while enhancing glutamine metabolism via GLS-mediated conversion to α-KG for the TCA cycle. ID also promotes lipid accumulation by activating SREBP-1c, which upregulates fatty acid synthesis genes (ACLY, ACC, FASN) and inhibits CPT1-mediated β-oxidation. These adaptations—together with inflammation-driven lipid storage—support tumor growth and survival under iron-deficient conditions.

ID profoundly impacts tumor cell proliferation and significantly alters their metabolic pathways, leading to adaptive changes in tumor metabolism. To survive in a low-iron environment, tumor cells undergo metabolic reprogramming to counteract iron-dependent metabolic disruptions (Figure 3). The effects of ID on tumor cell metabolism are discussed in the following sections.

### 4.1 Inhibition of mitochondrial function and oxidative phosphorylation

Mitochondria are organelles chiefly responsible for oxidative phosphorylation, converting oxygen and nutrients into cellular energy. They also play key roles in heme production and in the assembly of iron-sulfur (Fe-S) protein clusters in eukaryotic cells (58). Iron is crucial for mitochondrial oxidative phosphorylation, and ID significantly reduces the activity of several iron-dependent enzymes involved in the electron transport chain, such as cytochrome c oxidase and succinate dehydrogenase (59). ID has been shown to impair mitochondrial function by increasing oxidative stress and causing damage to mitochondrial DNA. Consequently, ID directly compromises mitochondrial efficiency, leading to a decline in oxidative phosphorylation. In the context of ID, the mitochondria in tumor cells cannot fully sustain energy production, resulting in reduced ATP synthesis and limited cellular energy metabolism.

### 4.2 Metabolic reprogramming and the “Warburg effect”

To compensate for impaired mitochondrial oxidative phosphorylation, tumor cells under ID conditions often shift to glycolysis for energy production, known as the “Warburg effect” (60). Although glycolysis is less efficient than oxidative phosphorylation, its upregulation allows tumor cells to produce ATP rapidly (Figure 2). The role of HIF-1α in this glycolytic shift is particularly critical. HIF-1α activation not only regulates the expression of glycolysis-related enzymes, such as hexokinase and lactate dehydrogenase but also enhances the expression of glucose transporters (GLUT), thereby increasing glucose uptake by tumor cells (51). Concurrently, to ensure adequate glucose supply to peripheral tissues, ID can elevate gluconeogenic markers such as lactate and alanine—reflecting increased glycolysis, impaired muscle oxidative metabolism, and enhanced protein catabolism—as well as catecholamine levels, which further stimulate hepatic gluconeogenesis (61, 62). This metabolic adaptation enables rapidly proliferating tumor cells to acquire energy independently of oxidative phosphorylation.

ID significantly impacts cellular energy metabolism by modulating key signaling pathways such as AMP-activated protein kinase (AMPK) and the mechanistic target of rapamycin (mTOR) (63, 64). AMPK, a cellular energy sensor, is activated under energy stress caused by ID-induced mitochondrial dysfunction and reduced ATP production. Activated AMPK inhibits mTOR signaling, promoting catabolic processes (e.g., autophagy, fatty acid oxidation) while suppressing anabolic processes (e.g., protein synthesis, cell growth) (64). In tumor cells, this AMPK-mediated mTOR inhibition limits proliferation by restricting energy and biosynthetic precursors (65).

In addition to glycolysis, the metabolic reprogramming induced by ID also affects glutamine metabolism (Figure 2). Studies have shown that under iron-deficient conditions, tumor cells increase glutamine uptake and metabolism to replenish intermediates in the tricarboxylic acid cycle, supporting continued cell growth (66). As a critical carbon and nitrogen source in metabolic reprogramming, glutamine is essential in energy production and antioxidant defense. ID may heighten tumor cells' reliance on glutamine to manage metabolic stress and sustain energy supplies. ID significantly affected glutamine metabolism by regulating HIF-1α and AMPK signaling pathways (Figure 3). HIF-1α up-regulates the expression of Glutaminase (GLS) to promote the decomposition of glutamine into glutamate, which is further converted to α-ketoglutarate (α-KG), which enters the tricarboxylic acid cycle (TCA cycle) to support energy production (67). In the tumor microenvironment, this metabolic adaptation likely enables tumor cells to continue growing under adverse conditions, suggesting that glutamine metabolism may serve as a critical compensatory mechanism in iron-deficient states.

Metabolic adaptations induced by ID aid in tumor cell survival and drive their progression. Research has demonstrated that ID enhances tumor cell invasiveness through metabolic reprogramming (68). In response, tumor cells upregulate glycolysis and glutamine metabolism to maintain energy production while simultaneously generating essential precursors for DNA synthesis, thus supporting rapid tumor proliferation and metastasis (69).

### 4.3 Alterations in lipid metabolism

Tissue ID affects the sympathetic nervous and endocrine systems, weakening the body's ability to respond to cold exposure and regulate thermogenesis. Brown adipose tissue (BAT), a specialized form of adipose tissue, can uncouple cellular respiration from ATP production. In humans, the activity of BAT shows an inverse relationship with levels of adiposity, blood glucose, and insulin sensitivity (61, 70). ID also significantly affects lipid metabolism in tumor cells—impaired mitochondrial function and reduced oxidative phosphorylation impact glucose metabolism and fatty acid oxidation (71).

Stabilization of HIF-1α further upregulates the expression and activity of SREBP-1c, converting SREBP-1c from an ER precursor to an active form in the nucleus and activating transcription of genes involved in fatty acid synthesis, such as ATP citrate lyase (ACLY), acetyl-coa carboxylase (ACC), and fatty acid synthase (FASN) (72). In addition, SREBP-1c activation also inhibits carnitine palmitoyltransferase 1 (CPT1) through the generation of Malonyl-CoA, thereby inhibiting fatty acid entry into mitochondria for β-oxidation, which further leads to lipid accumulation in the cell (Figure 3). Under iron-deficient conditions, fatty acid oxidation is inhibited, leading tumor cells to accumulate lipids by upregulating fatty acid synthesis (73). This process provides materials for cell membrane construction and helps manage oxidative stress through lipid metabolism regulation. Additionally, oxidative stress resulting from ID elevates pro-inflammatory cytokine levels, further promoting lipid accumulation by activating white blood cells (74).

### 4.4 Interaction between oxidative stress and ID

Oxidative stress is a significant component of the metabolic disruptions caused by ID. In ID conditions, oxidative stress markers such as Cu/Zn-superoxide dismutase (Cu/Zn-SOD) increase, while markers of antioxidant defense decrease (22, 75). For instance, levels of enzymatic and non-enzymatic antioxidants—including catalase (CAT), superoxide dismutase (SOD), glutathione peroxidase (GSH-Px), and vitamins A, C, and E—are reduced during ID (22, 74, 76, 77). These shifts increase ROS production and a decline in the body's overall antioxidant capacity. Prolonged ID weakens cellular antioxidant defenses, making tumor cells more vulnerable to oxidative stress damage. Excessive ROS can lead to cellular damage and DNA mutations, contributing to tumor cell growth, metastasis, and resistance to treatment (78). The elevated ROS from oxidative stress may also cause hemoglobin to release heme groups, leading to circulating free heme, which triggers further free radical production and exacerbates cellular damage (22).

## 5 ID and the tumor microenvironment

### 5.1 The effect of ID on the tumor immune microenvironment

**Figure 4:**
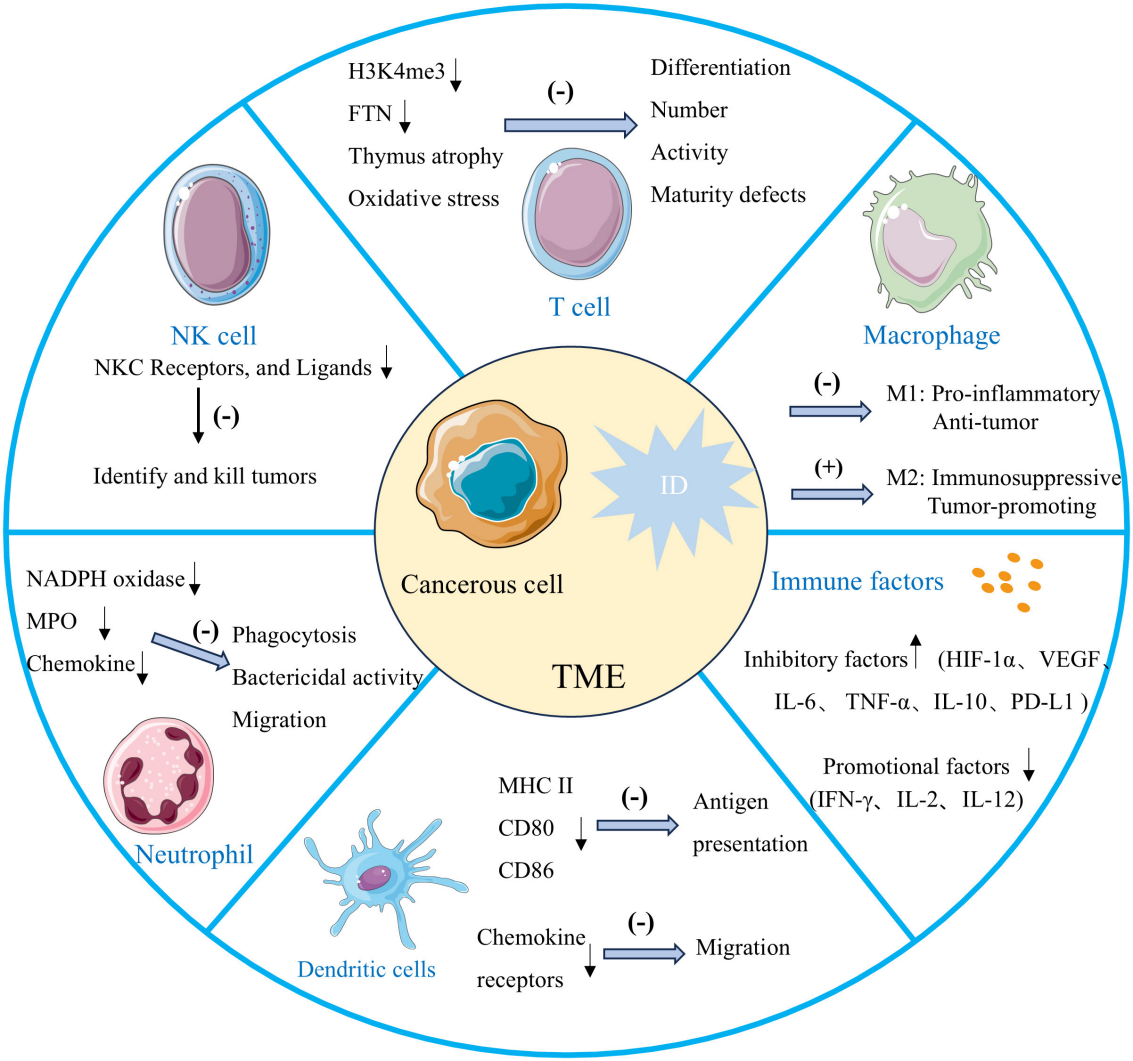
The effects of ID within the tumor microenvironment on immune system function. ID weakens antitumor immunity by impairing NK cell cytotoxicity, skewing macrophages toward immunosuppressive M2 phenotypes, reducing T cell proliferation, differentiation, and inhibiting dendritic cell migration and neutrophil function. It also alters immune factors, increasing inhibitory mediators and reducing antitumor-promoting factors. Together, these changes create an immunosuppressive microenvironment that promotes tumor growth and progression.

Iron is essential for immune cell function, which is critical in regulating the growth and differentiation of immune cells to maintain a robust immune system. ID can weaken immune responses, reduce immune surveillance and alterations in the tumor immune microenvironment, and possibly facilitate cancer progression (79). Additionally, iron is necessary for the regular activity of peroxidase and synthetase enzymes involved in nitric oxide production, which is crucial for sustaining immune cell function (61, 80). Changes in iron metabolism significantly impact the regulation of various immune cells in the tumor microenvironment, particularly natural killer (NK) cells, T cells, macrophages, neutrophils, and various immune factors (Figure 4). ID reduces the antitumor effectiveness of NK cells by promoting hypoxic conditions (79). NK cells are cytotoxic effector lymphocytes with critical roles in innate immunity, including immune surveillance and antitumor activity. NK cells kill tumor cells by releasing cytotoxic molecules such as perforin and granzyme (81). ID can suppress the expression of receptors and NK cell ligands on tumor cell membranes that are essential for NK cell activation (22, 82). ID compromises NK cells function by limiting their production of cytotoxic molecules, thereby reducing their tumoricidal capacity. Consequently, ID disrupts NK cells' cytotoxic and specific antitumor functions, promoting tumorigenesis and tumor growth. ID reduces the production of IFN-γ, a critical cytokine secreted by NK cells that activates other immune cells and boosts tumor cell destruction (83). This decrease in IFN-γ production weakens the immune response, further impairing NK cells' ability to control tumor growth (84).

ID can profoundly influence macrophage polarization. Research indicates that iron-deficient conditions may skew macrophages toward either M1-type activity, impairing their pro-inflammatory and antitumor functions, or toward M2-type polarization, typically associated with immunosuppressive properties. M1 macrophages depend on iron for producing ROS, which are crucial for their ability to kill pathogens and tumor cells. ID reduces ROS production, weakening M1 macrophages' capacity to target and destroy cancer cells, thus impairing the body's antitumor immune response (85). M2 macrophages can facilitate tumor growth and metastasis through the secretion of anti-inflammatory factors like IL-10 (79, 86). Additionally, they support tumor progression by enhancing angiogenesis through the release of factors like VEGF, improving the blood and nutrient supply to the tumor (87). Furthermore, in an ID state, the iron transport and storage functions of M2 macrophages are altered, resulting in reduced iron availability within the tumor microenvironment, further exacerbating immunosuppression and supporting tumor progression. Notably, in tumors with a high abundance of pro-tumoral M2 macrophages—the principal iron recyclers—ID may transiently restrict extracellular iron availability by limiting macrophage-mediated iron release, thereby potentially reducing tumor cell proliferation. However, this potential inhibitory effect can be counterbalanced by the fact that ID itself promotes M2-like polarization, further enhancing the secretion of immunosuppressive cytokines and pro-angiogenic factors. This dual role illustrates the context-dependent nature of ID in tumor progression and highlights the importance of considering the immune composition of the tumor microenvironment when evaluating iron-targeted therapeutic strategies.

T cells, as the central players in antitumor immune defense, rely heavily on iron-supported cellular metabolism for their proper function (88, 89). Iron is essential for mitochondrial ATP production, which provides the necessary energy for T cells to rapidly proliferate and activate upon encountering tumor cells. When iron supply is insufficient, mitochondrial function is impaired, leading to reduced ATP generation. This energy deficiency directly limits T cell proliferation capacity, thereby weakening their ability to effectively respond to tumor cells and other immune challenges (90). Histone demethylation, crucial for T cell differentiation, relies on the iron-dependent enzyme histone demethylase to demethylate H3K4me3 (trimethylation of histone H3 at lysine 4). ID can thus impact T lymphocyte differentiation by limiting this demethylation process (91). Studies show that ID decreases T cell proliferative capacity by reducing iron storage protein FTN levels. This leads to defective T cell maturation and impaired effector function, resulting in fewer circulating T cells (91). ID impairs the differentiation of Th17 cells, which are crucial in immune responses (81). Th17 cells secrete cytokines such as IL-17 to coordinate inflammation and immune defense, playing a particularly important role in antitumor processes. Impaired Th17 cell differentiation weakens the body's immune defense capacity (92). Consistently, ID also causes atrophy of the thymus, the primary site of T cell maturation (93). Additionally, ID significantly reduces CD4+ T cell proliferation and cell cycle progression, contributing to the reduced number of circulating T cells (94). Increased oxidative stress associated with ID further impairs T-cell activity (79). ID inhibits T cells from producing IFN-γ, which enhances the immune system's ability to attack and eliminate tumor cells (95). This cytokine is crucial for the cytotoxic activity of T cells, enabling them to effectively recognize and kill tumor cells. When iron levels are insufficient, the production of IFN-γ decreases, weakening the antitumor response of T cells and thus reducing the efficacy of immunotherapies such as immune checkpoint inhibitors (96). Therefore, ID has widespread and profound effects on T cells, including disrupting energy metabolism, inhibiting the expression of differentiation-related genes, and reducing the production of key cytokines, thereby impairing the immune control over tumors and diminishing the overall effectiveness of immunotherapy.

ID also inhibits the maturation of dendritic cells, compromising their ability to activate and stimulate immune responses. Iron plays a key role in the differentiation of dendritic cells by supporting the induction of the cyclin-dependent kinase inhibitor p21 (79). Under iron-deficient conditions, dendritic cells exhibit reduced expression of surface markers such as MHC II, CD80, and CD86, which diminishes their capacity to present antigens to T cells (97). Additionally, ID impairs the migration of dendritic cells to lymphoid tissues by altering chemokine receptor expression and reducing their responsiveness to chemical signals. ID also significantly reduces the production of IL-2 and IFN-γ (98). It is also known that myeloperoxidase (MPO) and NADPH oxidase are enzymes that play a vital role in the induction of IFN-γ by monocytes and in the killing and phagocytosis of microorganisms through the production of ROS by neutrophils and that these enzymes are iron-dependent, and that their catalytic activity is inhibited by ID, leading to impaired phagocytosis, reduced bactericidal activity, and induced tumor emergence (99, 100). At the bone marrow level, neutrophil production appears more iron-dependent than other leukocytes, and ID disrupts neutrophil production. Additionally, ID affects chemokine production and receptor expression, reducing neutrophil chemotaxis and impairing their migration to infection sites (93, 101). ID creates an immunosuppressive microenvironment conducive to tumor growth by impairing the function of immune cells such as NK cells, macrophages, and T cells, along with various immune-related factors and enzymes (102, 103).

In a healthy body, immune cells conduct immune surveillance, detecting and eliminating potentially transformed cells before they develop into malignant tumors. Iron is essential for this process, as it supports immune cell growth and differentiation and modulates cell-mediated immune responses and cytokine activity. Consequently, ID may impair the immune surveillance response, enabling tumor cells to evade immune detection and potentially leading to insufficient tumor cell elimination (79, 104).

Several mechanisms link ID to immune escape in tumors. First, ID impairs immune cell function, allowing tumor cells to evade immune recognition and attack. ID results in an undifferentiated phenotype lacking dendritic projections in dendritic cells, which are essential for T cell stimulation. This impairs T cell cytotoxicity, reducing their ability to kill tumor cells. Similarly, ID decreases T cell motility by inhibiting protein kinase C (75). Second, ID promotes the polarization of macrophages into the M2 phenotype, which creates an immunosuppressive environment by secreting anti-inflammatory factors and directly inhibits T cell activity through interactions with T cells. Additionally, ID causes the degradation of granzyme B, an enzyme derived from NK cells that is critical for cancer cell elimination. Consequently, NK cells in iron-deficient patients may exhibit reduced immune surveillance capacity, enabling cancer cells to evade immune destruction (96). Additionally, ID may impact antigen presentation on the surface of tumor cells. Studies have shown that ID reduces the expression of primary histocompatibility complex class I (MHC-I) molecules on tumor cell surfaces, diminishing the immune system's ability to recognize tumor cells (105). This enables tumor cells to “hide” from the immune system, evading immune attack. Furthermore, hypoxia and metabolic reprogramming induced by ID contribute to immune escape mechanisms. For instance, HIF-1α activation enhances tumor cell survival and further suppresses the host's antitumor immune response by impairing immune cell function (106).

ID modulates immune checkpoint pathways, facilitating tumor immune escape through multiple mechanisms. ID stabilizes HIF-1α, which transcriptionally activates PD-L1 in tumor cells. Increased PD-L1 expression suppresses T-cell effector functions by binding to PD-1 on T cells, thereby promoting immune evasion (107). Additionally, ID enhances the immunosuppressive activity of regulatory T cells (Tregs) by upregulating CTLA-4 expression. ID-induced oxidative stress activates the STAT5 pathway, which further promotes CTLA-4 expression on Tregs, inhibiting CD8+ T-cell proliferation and antitumor responses. Beyond individual checkpoint molecules, ID has also been implicated in immune checkpoint inhibitor (ICI) resistance by reducing CD8+ T cell infiltration and impairing their effector functions, which may compromise ICI efficacy. These mechanisms collectively contribute to an immunosuppressive tumor microenvironment, enabling tumors to evade immune surveillance and sustain progression.

### 5.2 Angiogenesis and hypoxia

Tumor growth relies on angiogenesis, and ID regulates this process through the HIF-1α pathway (108). HIF-1α is post-transcriptionally regulated by prolyl hydroxylase (PHD), an iron-dependent enzyme responsible for its degradation. Therefore, ID impairs PHD activity, leading to the accumulation of HIF-1α (109). Under low iron conditions, HIF-1α degradation is impaired, leading to increased stability. HIF-1α activation induces the expression of VEGF and other pro-angiogenic factors, promoting neovascularization. VEGF, in particular, is a potent driver of malignant angiogenesis and metastasis (110). However, these new blood vessels are often structurally abnormal and functionally inefficient, exacerbating local hypoxia within the tumor. Elevated VEGF levels, observed in breast and renal cell carcinomas, are associated with poor prognosis (111, 112).

Nuclear factor kappa B (NF-κB), a key transcription factor, plays a pivotal role in regulating immune responses. ID triggers oxidative stress and inflammation, both of which can activate the NF-κB signaling pathway. NF-κB activation allows tumor cells to evade growth-inhibitory signals, stimulate angiogenesis, invade tissues, metastasize, and replicate indefinitely (113, 114). Therefore, ID may weaken immune responses and increase the risk of tumorigenesis, contributing to poor prognosis and treatment resistance in cases of malignancy.

Under the combined influence of hypoxia and ID, tumor cells adjust metabolic reprogramming-related target genes via the HIF-1α pathway, promoting cancer progression (115, 116). These cells rely on glycolysis for energy production, leading to lactic acid accumulation, which exacerbates the acidic state of the tumor microenvironment (66). This hypoxic and acidic environment not only facilitates tumor invasion and metastasis but also enhances tumor resistance to anticancer therapies.

## 6 The role of ID and ferroptosis in tumors

### 6.1 Ferroptosis mechanism

Ferroptosis is a form of iron-dependent, non-apoptotic cell death marked by the buildup of lipid peroxides, which are catalyzed by iron. This process ultimately results in damage to the cell membrane and cell death (117). Unlike traditional programmed cell death forms, such as apoptosis and necrosis, ferroptosis is primarily regulated by iron and lipid metabolism. Iron generates excess ROS through the Fenton reaction, which directly acts on cell membranes rich in polyunsaturated fatty acids (PUFAs) to induce lipid peroxidation (118).

In ferroptosis, GPX4 is a critical inhibitor that prevents lipid peroxidation accumulation by reducing lipid peroxides. GSH, a cofactor of GPX4, is directly involved in the antioxidant response, helping to maintain cell membrane integrity (119). When GSH levels are reduced or GPX4 function is impaired, cellular antioxidant capacity is weakened, accumulating iron-dependent lipid peroxidation and triggering ferroptosis (118, 120). Beyond the GSH/GPX4 system, ferroptosis regulation involves other critical molecules in lipid and iron metabolism. For instance, FTN light and heavy chains are essential for iron storage and maintaining intracellular iron homeostasis. Dysregulation of FTN during ferroptosis can result in iron overload, further exacerbating lipid peroxidation.

### 6.2 How ID affects ferroptosis

While iron is a crucial driver of ferroptosis, ID can modulate susceptibility to ferroptosis, particularly in tumor cells. ID reduces ferroptosis by lowering the intracellular iron levels that drive this process (64, 121). On the other hand, ID can also impair the activity of antioxidant enzymes such as glutathione peroxidase (GPX4), which relies on glutathione (GSH) to neutralize lipid peroxides and prevent ferroptosis. For example, studies have found that ID and IDA lead to a significant reduction in GSH-Px (a member of the GPX family) and GSH-R activities, indicating a compromised antioxidant defense system (122). This reduction in antioxidant capacity could theoretically increase susceptibility to ferroptosis due to the accumulation of lipid peroxides. However, the overall effect of ID on ferroptosis in tumor cells may depend on the balance between reduced iron availability (which inhibits ferroptosis) and impaired antioxidant defenses (which could promote ferroptosis).

Although the mechanisms of ferroptosis in tumors have been preliminarily elucidated, it remains unclear whether ID may influence tumor progression through other non-ferroptosis pathways (108). For instance, the effects of ID on tumor cells' metabolic adaptability and immune evasion mechanisms are not yet fully understood (123). As research into ferroptosis mechanisms advances, drugs and compounds that induce ferroptosis are anticipated to offer therapeutic benefits against tumors, particularly those resistant to traditional apoptotic pathways (14). Addressing these open questions through in-depth studies will enhance our understanding of the role of iron metabolism in tumorigenesis, tumor progression, and treatment. Advancing research on ferroptosis mechanisms and iron regulation, particularly in tumors resistant to conventional therapies, holds promise for ferroptosis inducers to emerge as breakthrough treatments.

## 7 Clinical significance and therapeutic strategies for ID

### 7.1 Clinical evidence for ID in tumor progression

Recent clinical studies increasingly indicate a close association between ID and the development and progression of various tumors. A single-center study involving patients with solid tumors and hematological cancers found that 42.6% of subjects were iron-deficient, while 33% were anemic. The tumors with the highest rates of ID were pancreatic (63%), colorectal (52%), and lung cancers (51%) (13, 124, 125).

ID and anemia remain significant public health challenges, with substantial variations across regions and populations. In 2016, anemia affected approximately 41.7% of children under 5 years, 40.1% of pregnant women, and 32.5% of non-pregnant women globally (5). While the World Health Organization (WHO) estimates that 42% of childhood anemia and 50% of anemia in women can be addressed through iron supplementation, meta-analyses suggest that the actual contribution of ID to anemia may be lower, at 25% in children and 37% in women (126). This discrepancy highlights the methodological challenges in assessing ID prevalence, particularly in low- and middle-income countries (LMICs), where inflammation and limited access to advanced biomarkers (e.g., serum ferritin) complicate accurate diagnosis.

Regional studies provide valuable insights into ID prevalence. For instance, among children aged 6 months to 5 years, ID rates range from 10.6% in Colombia to 26.1% in Liberia, with intermediate values observed in Cameroon (20.2%), Laos (18.4%), and Mexico (14.8%). Similarly, among non-pregnant premenopausal women, ID prevalence varies from 13.7% in Cameroon to 30.4% in Mexico, reflecting the influence of socioeconomic and dietary factors (127). In high-income countries such as the United States, ID remains a concern, particularly among vulnerable groups, with prevalence rates of 11% in children, 15% in premenopausal women, and 18% in pregnant women (5). Disadvantaged populations, including low-income individuals, Indigenous communities, and migrants from LMICs, bear a disproportionate burden of both ID and anemia.

Inadequate iron intake or reduced iron stores in the body have been linked to an elevated risk of gastrointestinal tumors (128). Plummer-Vinson syndrome, which is characterized by ID anemia, upper airway stenosis, and dysphagia, has been associated with an increased risk of developing gastric and esophageal cancers (129). ID is particularly prevalent among colorectal cancer patients and is associated with more aggressive tumors and poorer prognosis (79, 130). One study found that individuals with ID had a fivefold higher risk of developing gastrointestinal tumors compared to the general population, while those with IDA faced a 31-fold higher risk (22, 131). Compared to non-anemic patients, those with IDA also tend to have poorer prognosis, worse tumor staging, and lower disease-free survival rates (132). Furthermore, a study on hepatocellular carcinoma reported that ID decreased ENPEP expression and promoted angiogenesis and metastasis through the transcription factor SP1, providing additional insight into how ID may enhance hepatocellular carcinoma progression (133).

In lung cancer, clinical observations indicate that ID is closely associated with systemic inflammatory responses and tumor progression. The transcription factor nuclear factor-E2-related factor-2 (Nrf2) is a cellular sensor of oxidative stress. ID can activate Nrf2 signaling, leading to autophagy and oxidative stress responses. Activation of Nrf2 has been implicated in cancer development and is correlated with poor prognosis and reduced survival, particularly in tumor types like non-small cell lung cancer (134, 135). Additionally, research by Muka et al. suggests that high iron intake may reduce the risk of lung cancer (136).

ID is common among breast cancer patients and is associated with tumor progression and metastasis. Studies have shown that ID may increase the aggressiveness of breast cancer by altering immune responses and promoting angiogenesis within the tumor microenvironment (137). Research using mouse models has demonstrated that an iron-deficient diet leads to the stabilization of HIF-α, which plays a crucial role in the malignancy and aggressiveness of breast cancer (138).

Abnormalities in iron metabolism are emerging as a significant area of research in brain tumors. Although direct clinical studies on the role of iron in patient prognosis are limited, most published studies have indirectly investigated iron by analyzing genes and proteins associated with iron metabolism. For instance, the observed role of anemia in glioblastoma (GBM) suggests that anemic patients may have poorer prognoses than non-anemic patients (93). In cancer patients, the most common cause of anemia is FID. A high prevalence of ID, particularly FID, has been reported in neuroblastoma patients, where FID is a significant characteristic and an independent predictor of event-free survival. However, further research is needed to understand the long-term effects of iron supplementation on different types of ID in neuroblastoma patients (139). In addition, the severity of ID has been linked to tumor load and metastasis in nasopharyngeal carcinoma (140). Supporting this, an animal study showed that oral cancer developed earlier in iron-deficient rats than those with normal iron levels (141). These studies indicate that ID is not only associated with the development and progression of various tumors but may also contribute to malignancy by altering the tumor microenvironment and modulating tumor cell metabolism.

Notably, the incidence and functional consequences of ID vary by tumor type and are shaped by tumor-intrinsic biology and the local immune landscape. Clinical series and recent reviews show high ID prevalence in cancers associated with chronic blood loss or systemic inflammation, where hepcidin-driven iron sequestration and impaired absorption commonly cause functional or absolute ID (142, 143). By contrast, immunologically “cold” tumors (characterized by low T-cell infiltration and limited inflammatory signaling) may be relatively less affected by ID through immune-mediated mechanisms, because their progression depends less on iron-sensitive immune surveillance (144, 145). Nevertheless, ID can still modulate cold tumors indirectly via effects on stromal cells, angiogenesis, and tumor metabolic reprogramming (e.g., HIF signaling, glycolysis). The net effect of ID—whether protective or permissive—thus remains tumor- and context-dependent. Therefore, we recommend tumor-specific assessment, integrating systemic iron indices with tumor and TME iron-handling markers and immune phenotype, to guide iron-targeted interventions in cancer patients.

In clinical and *in vivo* contexts, ID in cancer patients can result from both systemic host factors and tumor-specific mechanisms. Systemically, chronic inflammation, malnutrition, reduced gastrointestinal absorption, and anemia of chronic disease can limit iron availability; inflammatory cytokines (e.g., IL-6) increase hepcidin expression, reducing intestinal iron absorption and iron release from macrophages (146, 147). At the tumor level, high iron demand due to rapid proliferation, upregulation of transferrin receptor 1, and downregulation of ferroportin promote iron sequestration within tumor cells, reducing circulating iron (41, 137). Current evidence indicates that these mechanisms can coexist, with their relative contribution varying by tumor type, disease stage, and patient status. Understanding this interplay is essential for interpreting ID in oncology and for designing iron-targeted interventions.

ID significantly impacts the prognosis of cancer patients, with studies indicating that it is associated with poorer survival outcomes and higher tumor recurrence rates (68, 148). ID has also been linked to the development or recurrence of specific cancers, such as gastrointestinal, lung, genitourinary, and premenopausal breast cancers. Mild to severe chemotherapy-induced anemia (Hb levels < 10 g/dl) has been reported in patients with solid tumors, including those of the breast, colon, rectum, lungs, ovaries, and stomach (13, 149–151). For example, low serum iron levels in breast and colorectal cancer patients are often associated with poorer treatment responses and a heightened risk of recurrence (132). A large-scale clinical study found that ID affects tumor growth and may reduce patient survival by impairing immune cell function and weakening the immune system's antitumor response (79, 152). A separate study found that breast and colorectal cancer patients with preoperative anemia who received FOLFOX adjuvant chemotherapy had a poorer prognosis compared to those without anemia. Furthermore, a systematic review of 60 studies found that anemic cancer patients faced a 65% higher overall risk of death compared to non-anemic patients (152, 153).

ID can reduce treatment effectiveness for patients undergoing radiation or chemotherapy. Studies have shown that ID weakens the response to these therapies, making patients more susceptible to complications such as fatigue, immunosuppression, and anemia. Additionally, ID is associated with resistance to anticancer drugs and may enhance tumor cell resistance through metabolic reprogramming and activation of anti-apoptotic signaling pathways (137). For instance, ID induces resistance to sorafenib in hepatocellular carcinoma cells by inhibiting apoptosis through upregulation of HIF-1α (154). Beyond its impact on treatment resistance and tumor progression, ID also plays a critical role in the development and exacerbation of cancer-associated cachexia and malnutrition.

ID and cancer-associated cachexia are bidirectionally linked through shared pathophysiological mechanisms. In cachexia, systemic inflammation elevates hepatic hepcidin, reducing intestinal iron absorption and sequestering iron in macrophages, thereby limiting iron availability for erythropoiesis and mitochondrial oxidative metabolism in skeletal muscle (146, 155, 156). This impairment decreases ATP production, promotes muscle weakness, and accelerates proteolysis (157). Malnutrition often coexists with cachexia in advanced-stage cancer and is linked with worse outcomes, including reduced chemotherapy efficacy and survival (158). Conversely, ID can intensify cachexia by aggravating fatigue, lowering physical activity, impairing protein synthesis, and contributing to anorexia and altered taste perception, ultimately worsening malnutrition. Evidence from cachectic models demonstrates that iron supplementation can preserve muscle mass and function, highlighting the mechanistic role of iron in mitigating cachexia (159). This vicious cycle underscores the importance of early detection and combined nutritional and iron-targeted interventions in advanced cancer care.

### 7.2 Treatment of ID

Dietary iron is obtained from heme sources—such as red meat, poultry, and fish—and non-heme sources—such as legumes, leafy greens, nuts, and fortified cereals (160). Heme iron is more bioavailable (15%−35%) and less affected by dietary inhibitors, while non-heme iron absorption is lower (2%−20%) but can be enhanced by vitamin C and inhibited by phytates, polyphenols, and calcium (161, 162). Adequate iron intake and optimal absorption are important for maintaining hemoglobin levels, supporting oxygen delivery, and enhancing the efficacy of chemotherapy and radiotherapy (163, 164). ID may impair treatment tolerance, exacerbate fatigue, and weaken immune responses, ultimately reducing therapeutic effectiveness. Nutritional strategies to optimize iron status may therefore contribute to improved outcomes in cancer patients.

Given the negative impact of ID on tumor progression and patient prognosis, iron supplementation therapy is gaining attention in the clinic. Appropriate iron supplementation therapy can improve the anemia status of cancer patients, improve quality of life, reduce fatigue, and enhance the response to chemotherapy and radiotherapy. However, iron supplementation therapy should still be used with caution in tumor patients, as excessive iron supplementation may promote tumor cell proliferation and metastasis.

There are several approaches to iron supplementation in iron-deficient patients, such as iron-fortified diets, oral iron supplementation, intravenous iron supplementation, erythropoiesis-stimulating agents (ESA), red blood cell transfusions (RBC), and iron metabolism modifying drugs (e.g., hepcidin inhibitors; Table 2). Oral iron is the treatment for asymptomatic or mildly symptomatic patients with FID or AID and no clinical or subclinical inflammation. However, oral iron therapy is ineffective in malignant disease because cancer is usually accompanied by inflammation, and intestinal iron absorption is significantly limited in cancer patients, with more than 95% of the iron excreted and the absorbed iron difficult to utilize efficiently for erythrocyte precursors. Thus, AID should always be addressed in cancer patients, whereas treatment for FID is necessary only when symptoms or severe anemia are present (8).

**Table 2:** Approaches to iron supplementation in ID patients.

**Type of intervention**	**Indications**	**Primary efficacy**	**Specificities**	**Side effects**	**Limitations**
Iron fortified diet	Mild IDA, or as a prophylaxis	Maintains basal iron levels and improves immunity	Natural source of food, suitable for long-term maintenance, convenient and easy to obtain	Slower effect, dependent on dietary absorption rate	Single dietary modifications may not be sufficient to address anemia, especially in patients with moderate to severe ID
Oral iron	Mild IDA or mild symptoms	Increases serum iron levels and relieves symptoms of mild anemia	Simple and easy to administer, suitable for long-term use, usually the treatment of choice	Gastrointestinal distress	Inefficient absorption and susceptibility to inhibition by tumor-induced inflammation
Intravenous iron	Moderate to severe anemia, ineffective oral iron, tumor or chemotherapy related anemia	Rapidly increases serum iron and hemoglobin levels and improves symptoms such as fatigue	Rapid effect, improves more severe anemia	Phlebitis, allergic reactions, iron overload risk	Higher cost and need to be operated in a medical setting
ESA	Patients with chemotherapy-associated anemia or need to enhance erythropoiesis	Relieves anemia by stimulating erythropoiesis and reduces the need for blood transfusions	Better efficacy in combination with iron supplementation for patients with moderate to severe anemia	Increased risk of thrombosis, especially in patients with tumors	Dependent on iron level support, limited effect alone
Combined treatment (iron + ESA)	Patients with chemotherapy-associated anemia or limited effect of ESA therapy alone	Enhances erythropoiesis and reduces the need for blood transfusions	Combined iron supplementation and ESA treatment works best to improve patient quality of life	Increased risk of thrombosis	Patient response to iron supplementation needs to be carefully monitored, with limited results in some patients
RBC	Patients with severe anemia or in urgent need of a rapid increase in hemoglobin levels	Immediately increases hemoglobin concentration and relieves symptoms of severe anemia	Rapid effect, suitable for acute anemia and pre-surgical patients	Transfusion reactions, risk of infection, immune rejection	Only short-term improvement of symptoms, the effect is not long-lasting, long-term use is more risky
Iron metabolism modifying drugs (e.g. hepcidin inhibitors)	Improvement of iron utilization by modulation of hepcidin	Increasing iron bioavailability by decreasing hepcidin	Direct modulation of iron metabolism, a prospective treatment for patients with specific ID	May cause liver function abnormalities, long-term safety not established	Mostly in the experimental research phase, lack of extensive clinical data

ID, iron deficiency; IDA, iron deficiency anemia; ESA, erythropoiesis-stimulating agents; RBC, red blood cell transfusions.

Blood transfusions should be administered cautiously due to potential complications, including infection, transfusion reactions, lung injury, alloimmunization, stroke, myocardial infarction, transmission of unidentified pathogens, thromboembolic events, renal injury, and cancer recurrence. These risks limit the broader use of blood transfusions; ESA may reduce the need for blood transfusions in cancer patients, but only 30%−75% of patients respond to ESA. Moreover, the use of ESA carries risks, such as a higher incidence of thromboembolic events when used off-label in patients not undergoing chemotherapy, as well as an increased risk of mortality (165). As a consequence, both the European Medicines Agency (EMA) and the U.S. Food and Drug Administration (FDA) have imposed stringent limitations on the use of ESA; Iron therapy for FID is a well-established treatment in nephrology, although its application in oncology is a more recent development (8).

To date, no international scientific organizations have released specific guidelines regarding iron supplementation for cancer patients. Nevertheless, certain institutions have provided recommendations for managing anemia associated with cancer or chemotherapy. The NCCN guidelines recommend oral or intravenous iron monotherapy for AID (FTN < 30 ng/ml, TSAT < 20%). When FTN levels are between 30 and 100 ng/ml and TSAT is between 20 and 50%, intravenous iron may be more effective than ESA use. In anemic patients with a TSAT < 20%, a combination of intravenous iron and ESA is recommended, but the use of intravenous iron in an infected state should be avoided; ASH/ASCO guidelines recommend monitoring iron homeostasis before and during ESA therapy and iron supplementation as needed to improve efficacy, but there is uncertainty about the optimal frequency of monitoring (165); ESMO and EORTC guidelines state that intravenous iron is more effective than oral iron in raising hemoglobin levels and reducing the need for blood transfusions and that intravenous iron can be used as a monotherapy in patients with Hb ≤ 11 g/dl, or Hb decrease by ≥2 g/dl and AID or FID and should be prioritized over ESA therapy (8, 166). In conclusion, intravenous iron has an essential role in the management of cancer or chemotherapy-induced anemia. However, treatment needs to be individualized, especially when there is a risk of infection.

For oral iron supplementation, increased iron intake can upregulate hepcidin—a key regulator of iron metabolism—which subsequently lowers plasma iron levels. In order to partially bypass this regulatory mechanism, an alternate-day regimen is recommended rather than daily dosing, thereby improving the overall effectiveness of oral iron supplementation (1, 167, 168). Although oral iron supplements are widely used for their accessibility and ease of administration, inflammatory signals in cancer patients may inhibit intestinal iron absorption, diminishing their efficacy. In light of this, intravenous iron supplementation is becoming increasingly prominent in iron replacement therapy for cancer patients (169).

Small studies have confirmed that intravenous iron alone increases Hb levels and reduces the need for blood transfusions (170). Modern iron agents such as ferric carboxymaltose responded well in patients with varying baseline iron levels, with fewer side effects and only 2.3% of patients experiencing minor discomfort (171). A combination of intravenous iron and ESA improves the hematologic response rate, shortens response time, and reduces transfusion requirements and ESA usage (172, 173). Combination therapy showed a significant response rate improvement in FID patients (34%−43%) and a lesser response rate improvement in non-FID patients (13%−19%) (8). The efficacy of intravenous iron is related to the total dose of iron, independent of the patient's initial iron level. Concomitant intravenous iron should be considered in patients with TSAT < 20% and FTN < 500 ng/ml who are scheduled to receive ESA therapy (8).

Intravenous iron has not been shown to increase the risk of infection or tumor growth, but it needs to be done by a medical professional to avoid rare side effects such as tissue necrosis. It should be avoided in cases of infection and ultra-high FTN levels. Intravenous iron is recommended for patients with FID or AID, especially before surgery or when receiving ESA therapy. Long-term intravenous iron therapy should be avoided in cancer patients because there is no safety data on the long-term use of iron for cancer treatment. ESA is indicated for patients with chemotherapy-associated anemia (Hb < 10 g/dl), and blood transfusions are limited to patients with life-threatening or severe symptoms. Studies confirm that iron supplementation is significantly associated with increased quality of life in tumor patients with ID in both the short and long term, and there are no studies on the effects of iron supplementation alone on patients on hormone therapy, targeted therapy, or immunotherapy (10).

## 8 Future research directions and challenges

Despite growing interest in the relationship between ID and tumorigenesis, many questions and significant research gaps still need to be addressed. First, the specific mechanisms by which ID affects different tumor types still need to be fully understood. The dual effects of ID are modulated by various factors, including tumor type, microenvironment, and individual patient differences, which require further investigation. Iron may exhibit both pro-tumorigenic and anti-tumorigenic effects across different cell types in the tumor microenvironment, making its overall impact on patient prognosis uncertain. Since interactions among multiple cell types influence prognosis, studying individual cell roles in isolation may not accurately capture the cumulative effect. For instance, iron might promote tumor cell proliferation while inhibiting migration or enhancing an antitumor T-cell response. This positive immune response could potentially offset the adverse effects of tumor cell proliferation driven by iron, highlighting the complexity of iron's role in cancer progression and patient outcomes.

Critical molecules in the iron metabolic pathway are emerging as potential targets for cancer therapy. Targeted approaches that modulate iron uptake, storage, and regulation are expected to open new avenues for tumor treatment. Studies indicate that high levels of TFR expression in many tumor types correlate with poor prognosis, and drugs targeting TFR1 may effectively disrupt the iron dependency of tumor cells, thereby inhibiting their growth (27, 137). Additionally, the roles of key molecules such as FTN and hepcidin in maintaining iron homeostasis have garnered significant attention, and modulating the expression or activity of these molecules may offer promising new therapeutic strategies for cancer (174, 175).

Future studies should incorporate multidisciplinary research from fields such as iron metabolism, bioinformatics, immunology, and oncology to fully elucidate the complex role of iron metabolism in tumors. The application of bioinformatics in cancer research—particularly in analyzing expression data of iron metabolism-related genes and proteins—can help identify new therapeutic targets and mechanisms. Bioinformatics tools enable a more precise examination of changes in iron metabolism networks across different tumor types, shedding light on the specific effects of ID within the tumor microenvironment. Simultaneously, the integrated study of immunology and oncology is essential. Recent research has shown that iron metabolism is closely tied to immune cell function, particularly regarding macrophages and T cells within the tumor microenvironment (110, 176). Future studies should further investigate how iron metabolism influences tumor immune evasion mechanisms and how the modulation of iron metabolism might enhance immunotherapy effectiveness. Additionally, the intersection of iron metabolism with broader metabolic processes in tumors is critical. Understanding how metabolic pathway alterations affect tumor cell survival and adaptability under iron-deficient conditions is an area that warrants deeper exploration (177).

## 9 Conclusions

The role of ID in tumorigenesis and progression is complex and multifaceted. While it can inhibit tumor cell growth and proliferation by limiting available iron, it may also promote tumor progression under certain conditions through mechanisms such as metabolic reprogramming, hypoxic responses, and immune evasion. ID may stabilize HIF-1α in specific tumor microenvironments, enhancing tumor angiogenesis and invasiveness. Furthermore, ID disrupts immune cell function, weakens antitumor immune responses, and contributes to tumor spread and treatment resistance. Although studies have highlighted the importance of iron metabolism disorders in various cancers, their specific mechanisms and roles in different tumor types remain unclear. Consequently, critical molecules in the iron metabolic pathway, such as FTN, TFR, and hepcidin, may become new targets for future tumor therapy. Looking ahead, tumor treatment strategies should further investigate the interrelationships between ID, the tumor microenvironment, immune regulation, and metabolic reprogramming. Multidisciplinary studies that integrate iron regulation with immunotherapy and metabolic interventions may pave the way for novel therapeutic approaches, ultimately improving patient survival and quality of life. Optimizing the dose and method of iron supplementation will be crucial for achieving precision treatment and minimizing side effects.

Future clinical studies will continue to explore the role of iron modulation in cancer therapy, aiming to provide cancer patients with more effective and safer treatment options.
